# An innovative diagnosis in gastrointestinal neuroendocrine neoplasms using Wax-Physisorption-Kinetics-based FTIR Imaging

**DOI:** 10.1038/s41598-022-22221-0

**Published:** 2022-10-13

**Authors:** Yi-Ting Chen, Pei-Yu Huang, Jaw-Yuan Wang, Yao-Chang Lee, Chee-Yin Chai

**Affiliations:** 1 Graduate Institute of Medicine, College of Medicine, Kaohsiung Medical University, Kao-hsiung, Taiwan; 2 Department of Pathology, Kaohsiung Medical University Hospital, Kaohsiung Medical University, No.100, Tzyou 1St Road, Kao-hsiung, 80708 Taiwan; 3 Department of Pathology, Faculty of Medicine, College of Medicine, Kaohsiung Medical University, Kao-hsiung, Taiwan; 4 Life Science Group, National Synchrotron Radiation Research Center, 101 Hsin-Ann Road, Hsinchu Science Park, Hsin-chu, 30076 Taiwan; 5 Division of Colorectal Surgery, Department of Surgery, Kaohsiung Medical University Hospital, Kaohsiung Medical University, Kao-hsiung, Taiwan; 6 Department of Surgery, Faculty of Medicine, College of Medicine, Kaohsiung Medical University, Kao-hsiung, Taiwan; 7 Graduate Institute of Clinical Medicine, College of Medicine, Kaohsiung Medical University, Kao-hsiung, Taiwan; 8 Center for Cancer Research, Kaohsiung Medical University, Kao-hsiung, Taiwan; 9 Department of Optics and Photonics, National Central University, Chungli, Taiwan; 10 Department of Chemistry, National Tsing Hua University, Hsin-chu, Taiwan; 11 Institute of Biomedical Sciences, National Sun Yat-Sen University, Kao-hsiung, Taiwan

**Keywords:** Imaging, Biological techniques, Cancer, Cancer imaging

## Abstract

Neuroendocrine neoplasm (NEN) is a common gastrointestinal (GI) tract tumor divided into the neuroendocrine tumor (NET) and neuroendocrine carcinoma (NEC) according to mitosis and Ki-67 index. However, the objective discordance between interobserver may cause unsuitable diagnosis and misleading treatment. Nowadays, aberrant glycosylation of glycoconjugates inducing further populations of elongated complex oligosaccharide covalent attached to glycoconjugates anchored in the cell membrane by neo-synthesis of cancer-associated alteration of carbohydrate determinants were observed during cancer development. This study aimed to demonstrate the wax physisorption kinetics coupled with Fourier transform infrared (WPK-FTIR) imaging between NET and NEC in the rectum, colon, and stomach by utilizing two wax reagents (beeswax and paraplast) as glycan adsorbents for physical binding glycans of glycoconjugates based on dipole-induced dipole interaction. Results showed greater physisorption with beeswax than that of paraplast, suggesting highly populated elongated glycans of glycoconjugates adhering onto the tumor surfaces of NETs than that of adjacent benign mucosa in the rectum and colon. Besides, the WPK results of gastric NEN tissue sections showed a higher infrared absorbance ratio of beeswax-remnant to paraplast-remnant remains onto the tissue sections referring to a higher population of elongated glycans in gastric NET as compared with that of gastric NEC. Based on our findings, different anatomical locations could share similar phenomena with minor variance. In conclusion, WPK-FTIR imaging may have the potential to be employed as an alternative diagnostic method in GI NENs in the future.

## Introduction

The histological definition of neuroendocrine neoplasms (NENs) arises from cells of the endocrine and nervous systems, producing and secreting polypeptide hormones, with the most common sites occurring in the gastrointestinal (GI) tract, lung, and pancreas^1^. In the digestive tract, the 2017 World Health Organization (WHO) classification scheme places neuroendocrine tumors into four main categories, including NET grade 1 (G1), NET G2, NET G3, and NEC (neuroendocrine carcinoma) G3, according to the mitotic activity and Ki-67 labeling index^1^. A higher grade of mitotic rate or Ki-67 index is used to define the final grade in cases where both markers are discordant.

According to the investigation of the Taiwan Cancer Registry between 1996 and 2008, a total of 2187 NEN cases were diagnosed, with a mean age of 57.9 years old and 62% males. The age-standardized incidence rate of NENs was 1.51 per 100,000 cases in 2008. The most common primary site was the rectum in Taiwan^2^. Clinically, patients with NET G1 usually have favorable prognosis after tumor excision. In contrast, fast growth and highly metastatic potential are characteristics of NECs, in which tumor volume is usually small but with aggressive metastasis before symptoms are diagnosed. Patients with NEC G3 decline rapidly as the disease progresses, then die due to distant metastases even after intensive surgical intervention and chemotherapy. Setting treatment strategies and predicting prognosis for NEN patients are strongly indicated by the results of histological categorization or dichotomization of tumor grade.

Currently, pathologists make the diagnosis based on morphology examination following WHO criteria; however, the mitotic count is affected by the size of the microscopic field or the number of neoplastic cells in a given area of the tumor tissue section, especially in limited specimens. The Ki-67 index also presents equivocal results predicting cell proliferation, especially in heterogeneous tumors. Well-differentiated morphology is based on typical organoid architecture, uniform nuclei, and coarse granulation, which may be influenced by fixation status or sampling factor. This indicates that pathological classification according to morphology, mitosis, and Ki-67 index is subjective due to interobserver variability, which can cause different final pathological diagnoses of grading.

Previous histopathological studies have shown that protein glycosylation, a post-translational modification process, is considered strongly related to cell proliferation, differentiation, and carcinogenesis^3–5^; furthermore, neo-synthesis and incomplete synthesis of glycans of glycoprotein have been proven during cancer development^6^. The glycosylation alteration is well-documented where malignant transformation and cancer progression result in fundamental changes in the glycosylation profile on the cell surface and secreted glycoproteins. Glycans of glycoconjugates play a crucial role in cell–cell communications, cell–cell recognition, cell–matrix adhesion, and cell differentiation^7^. Aberrant glycosylation associated with neo-synthesis and incomplete synthesis can cause elongated and branched protein-linked glycans of glycoprotein, especially in carcinogenesis^6,8^. Glycoconjugates are synthesized by glycosyltransferases and dynamically modulated by the concentration of glycosyltransferases and pH value of microenvironment^9^.

Fourier-transform infrared spectroscopy (FTIR) imaging has been utilized for differentiating malignant from normal specimens. The aberrant glycosylation of glycoconjugates anchored in the surface membrane of skin cancer cell samples, ovary cancer, and oral cavity cancer are successfully differentiated tumors from normal tissue sections with wax physisorption kinetics (WPK) and FTIR imaging^3–5^. In the WPK procedure, paraplast wax and beeswax employed as glycan adsorbents were proven to bind regular glycans (short-chain glycans) and elongated glycans (long-chain glycans) of glycoconjugates respectively. Furthermore, FTIR imaging is a state-of-the-art technique that reveals laterally-resolved chemical images of the distribution of paraplast wax and beeswax remnants respectively binding to regular chain and elongated glycans of glycoconjugates to differentiate normal from malignant. The greater the amount of glycan adsorbent remnant adhering onto the tissue section surface, the higher the population of glycans with similar chain length to glycans absorbance expected; and the amount of glycan adsorbent remnants can be utilized for correlating with the characteristic absorbance of glycan adsorbent in the spectral range of 3000–2800 cm^−1^. According to the principle of chemical similarity, if the geometric structure between molecules is similar, the physical adsorption between molecules is stronger. Therefore, if more glycan adsorbent remnant adhering to the sample surface is observed, the geometric structure of glycan adsorbent is expected to be similar to that of the glycan of glycoconjugate anchored in the sample surface.

WPK-FTIR imaging can provide infrared spectral images to unfold the distribution of regular and elongated glycans of glycoconjugates anchoring in the tissue section samples. Previously, this method has been proven to differentiate benign lesions from malignancy in the oral, ovary, and skin cancers^3–5^. Herein, we aimed to investigate the population of regular and elongated glycans of glycoconjugates anchored in the cell surface of G1 NETs and NECs tissue of the GI tract, as compared with that of the adjacent normal mucosa by utilizing two wax reagents with WPK-FTIR imaging. As we know, G1 NETs and NECs are the two ends of NENs. Besides NET G2 is relatively few in GI tract^10,11^ and NET G3 remains a gray zone between NEC. Therefore, G1 NETs and NECs were chosen for further evaluation for study, which may give us information if WPK-FTIR could be applied to neuroendocrine neoplasms. This method may have potential as a diagnostic tool for clinical diagnosis and decision-making for designing personalized therapeutic guidelines.

## Materials and methods

### Histological analysis of colorectal and gastric tissue sections

Six formalin-fixed, paraffin-embedded human GI NEN tissue sections, including every two samples (one NET and one NEC) of rectum, colon, and stomach were collected from the Kaohsiung Medical University Hospital. The study protocol complied with the ethical principles of the Helsinki Declaration and was approved by the Institutional Review Board of Kaohsiung Medical University Hospital (IRB No. KMUH-IRB-E-20150008). Specimens were prepared based on standard pathological procedures. Tissue sections of four-micron thickness were prepared and fixed on a MIR low-E slide (Kevley, Technologies, Chesterfield, OH, USA) for the inspection of WPK-FTIR imaging, and the hematoxylin and eosin (H&E) stained tissue sections were reviewed to confirm the diagnosis. The WHO grading system^1^ published in 2019 was applied to tumor grading. Half the tissue section samples were assigned to NET G1 and the other to NEC G3.

### Procedures of Wax Physisorption Kinetics and Chemical Image Construction of FTIR Imaging

Formalin-fixed, paraffin-embedded human NEN tissues fixed on a MIR Low-E slide were washed by soaking in a glass tank containing 30 ml xylene at 53 °C for 10 min to remove exited embedded paraffin within paraffin-embedded tissue sections for further processing. In WPK procedures, two wax reagents paraplast wax (Paraplast, tissue embedding medium, McCormick Scientific Leica) and beeswax were employed as the glycans adsorbents for binding regular glycans (short-chain type) of normal cells and elongated glycans (long-chain type) of cancer cells, respectively. Washed and dried tissue section samples were waxed by soaking in a glass tank filled with 30 ml wax-xylene solution for 2 min, consisting of 5.5 wt% of Paraplast wax or beeswax, and then xylene was evaporated at 53 °C for 10 min. Paraplast wax and beeswax with an aliphatic straight chain of cyanide (carbon–nitrogen, C.N. 30) were used as the glycan adsorbent for the normal glycans and the elongated glycan with increased terminal sialyation of the cancerous tissue section respectively. During the dewaxing process, tissue samples were waxed by soaking in a glass tank filled with 40 ml xylene at 53 °C for 5 s for dewaxing, and then the xylene remnant was evaporated on a hot plate at 53 °C for 2 min.

The FTIR imaging was performed using FT-IR imaging, including an FT-IR spectrometer (Nicolet 6700, Thermo Fisher Scientific, Madison, WI, USA) coupled with a confocal infrared microscope (Nicolet Continuum, Thermo Fisher Scientific) equipped with a 64 × 64-pixel liquid nitrogen-cooled mercury-cadmium-telluride detector, at BL14A1 of National Synchrotron Radiation Research Center (NSRRC) in Taiwan. Furthermore, the spectral images of the remaining glycan probe adhering onto mice GI tissue sections were acquired by using SR-IMS and established by integrating the characteristic absorption in the spectral range of 3000–2800 cm^−1^ and accumulating 64 scans at a spectral resolution of 4 cm^−1^ for the field of view 170 × 170 μm^2^ of each area of interest.

WPK-FTIR imaging was performed to evaluate the strength of physisorption interaction between wax adsorbent and glycans of glycoconjugates of tissue sections. Furthermore, the amount of wax remnant adhering to the surface of tissue sections after the waxing-dewaxing procedure was utilized to correlate with the IR absorbance in the spectral range of 3000–2800 cm^−1^ correlated with the strength of physisorption interaction between wax and glycans of glycoconjugates of tissue section surfaces. The greater absorbance of the remaining wax adsorbent was observed, and a stronger interaction was expected between wax adsorbent and glycan residue of glycoconjugates anchoring in the sample surface.

### Analysis of FTIR spectral data

**Figure 1:**
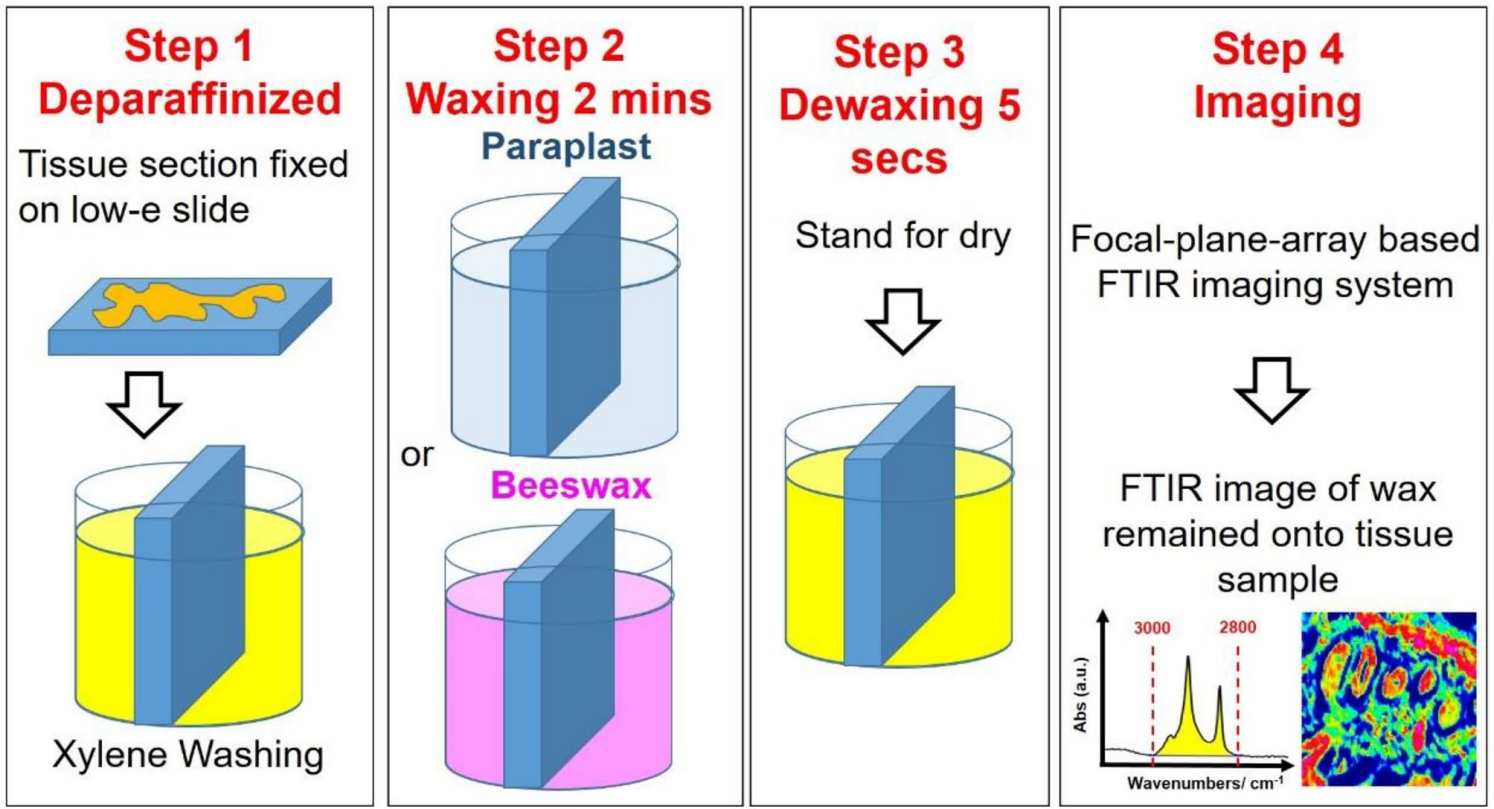
Process of WPK of wax residual imaging for NENs detected by FTIR.

The baseline-corrected and normalized FTIR spectra of tissue section samples were performed by using OMNIC™ (version 8.5; Thermo Fisher Scientific, Waltham, MA, USA). The amount of wax remnant adhering to the tissue section was utilized to correlate with the IR absorbance by integrating the characteristic absorption in the range of 3000–2800 cm^−1^ using OPUS (v. 6.5, Bruker Optics, Ettligen, Germany). Three areas of interest were investigated in the tissue sections for the presence of adjacent benign and malignant lesions after histopathological examination of all specimens. The wax remnant of the tissue section was measured based on the procedures of WPK-FTIR imaging as shown in Fig. 1.

Moreover, there were four normal alkanes (n-alkane, n-C_n_H_2n+2_) with different carbon–nitrogen (C.N.) as the wax adsorbents, employed as glycan probes, for targeting the glycan residues of glycoconjugates with similar chain lengths based on the chemical similarity principle. The chain length alteration of glycan residue of a glycoprotein can be normalized with the C.N. of the remaining n-alkane on the detection areas of interest in the GI tissue section surface. The reference image (Ref. Image) of the sample was defined as the IR absorbance of wax adsorbent before waxing. The raw data was defined as the IR absorbance data after waxing and dewaxing. The final IR absorbance indicating the remaining wax was calculated by subtracting (Sub.) reference data from raw data.

### Statistical analysis

All statistical analyses were performed with SPSS 19.0 (Chicago, IL, USA). Paired t-test was applied for correlation analysis. All tests were 2-sided, and a p-value less than 0.05 were considered statistically significant.

## Results

The wax remnant on the surface of the GI tissue section after the waxing-dewaxing procedure was investigated using WPK-FTIR imaging, utilized for profiling the population of the aberrant elongated glycan remnant of glycoconjugates anchored in the cell membrane based on the wax remnant with C.N. adhering to glycan of glycoconjugates anchored in the surface of tissue section sample.

### Neuroendocrine tumor in rectal tissues

**Figure 2:**
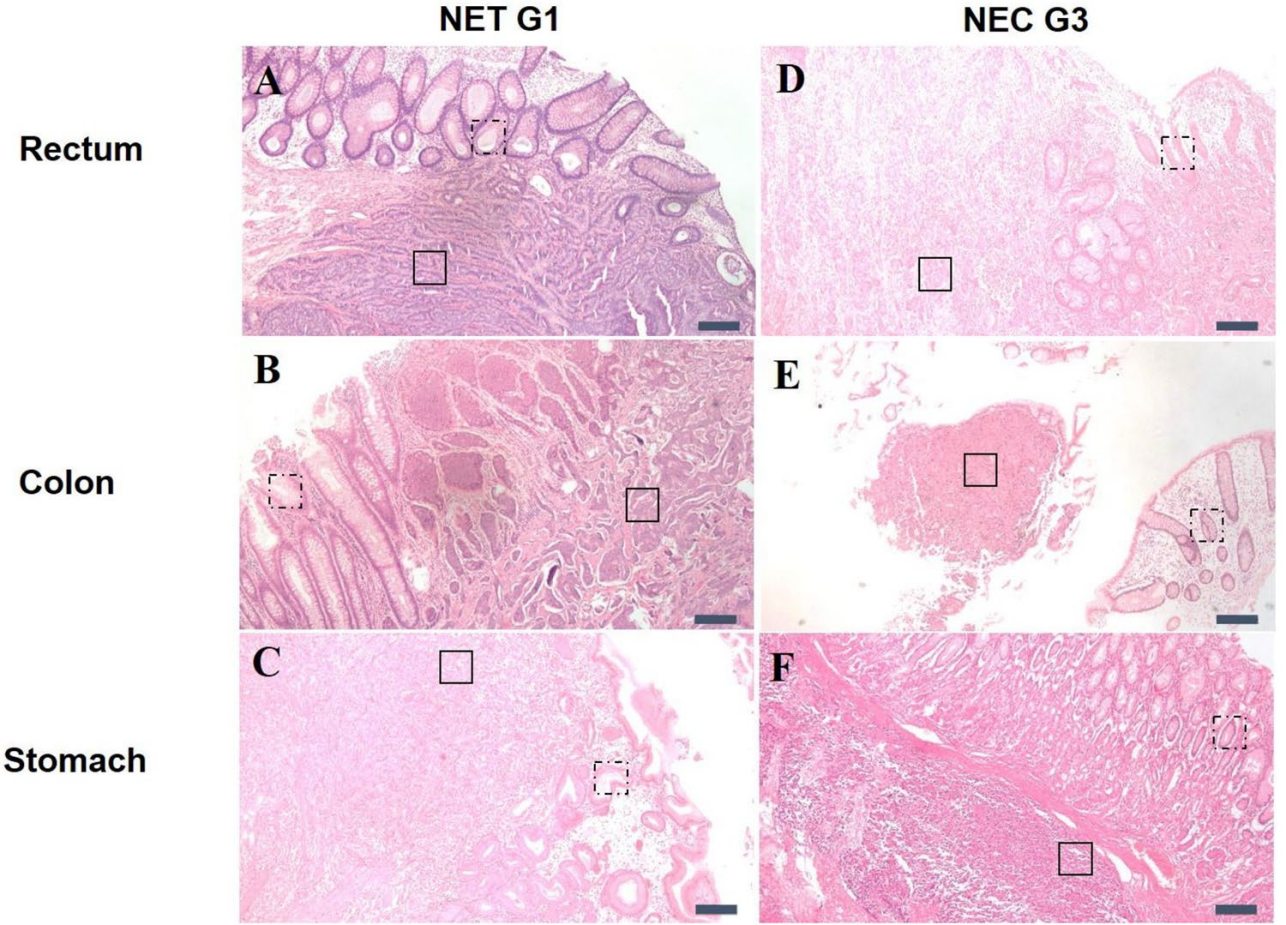
Representative findings of tumor (solid line) and adjacent normal mucosa (dotted line) of NET (**A**–**C**) and NEC (**D**–**F**) in histological sections. The scale bar indicates 200 μm.

Formalin-fixed paraffin-embedded human rectal NEN tissue sections of NET G1 and NEC G3 were investigated using WPK-FTIR imaging after histopathological examination as shown in Fig. 2A and D. We chose three representative locations of benign (adjacent normal mucosal epithelium) and malignant lesions in all specimens. The tumor cell nests were typically located in the submucosal area.

**Figure 3:**
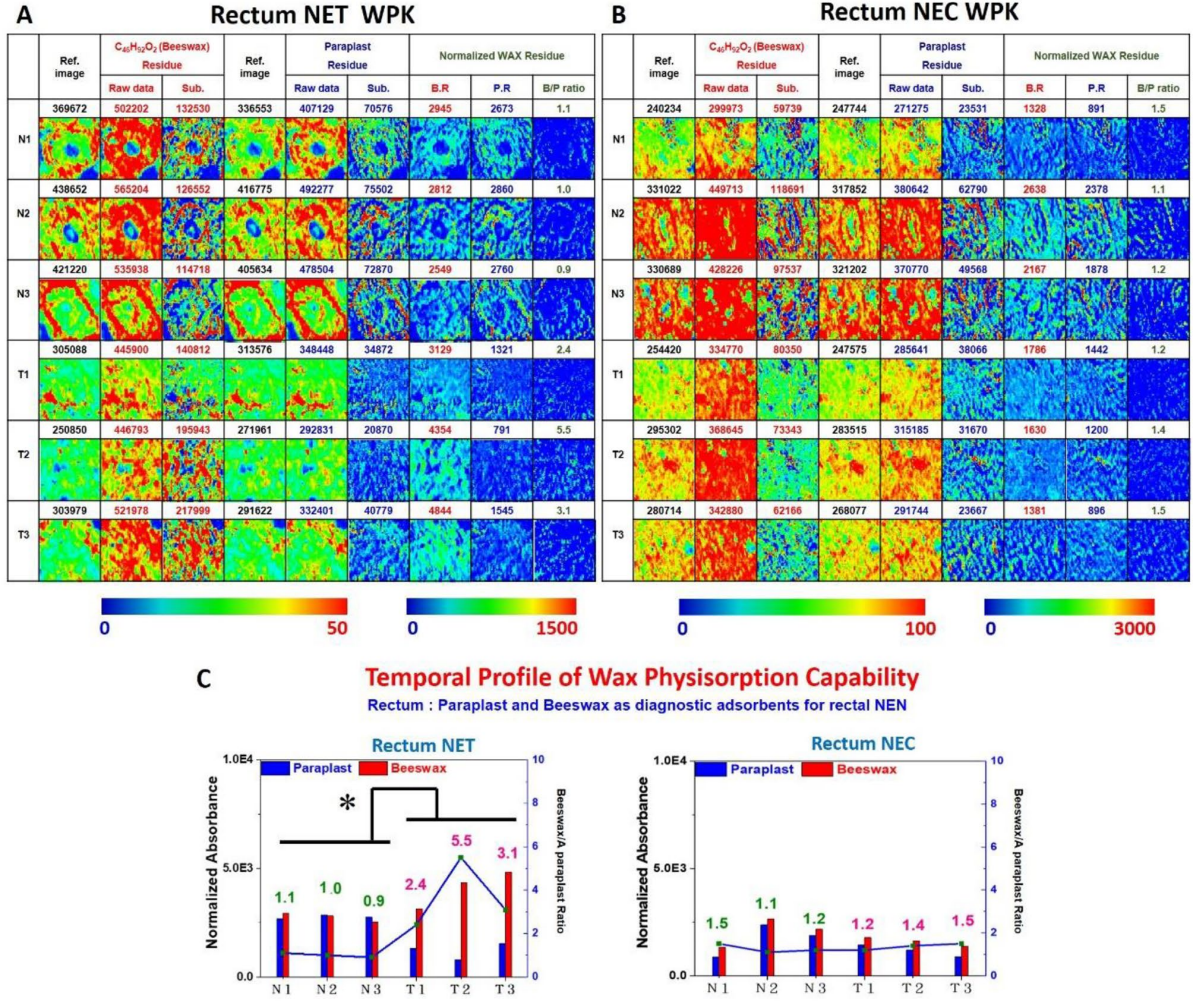
(**A**, **B**) The WPK-FTIR images present the distribution of remnants of beeswax and paraplast wax adhering to the surface of rectal tissue in NET and NEC. (Ref. Image: reference image before waxing, Raw data: the IR absorbance data after waxing and dewaxing, Sub: subtract reference data from raw data.) The left column of the images absorbance scale indicates the beeswax and paraplast absorbance before normalizing by the number of carbon atoms. The right column of the absorbance scale indicates the absorbance after normalizing. (**C**) The quantitative results and the ratio of average adsorption levels of wax normalized by the number of carbon atoms on human rectal NENs. (X axes: each three representative interest areas of adjacent normal mucosa (N) or tumor area (T); left Y axes: average adsorption levels of beeswax and paraplast wax; right Y axes and the number over the histograms: the ratio of beeswax /paraplast wax.) There was a significantly higher IR absorbance ratio of beeswax-remnant to paraplast- remnant between malignant and adjacent benign areas in rectal NET G1 (*p = 0.047).

The IR characteristic absorption of wax as glycan adsorbent in the spectral range of 3000–2800 cm^−1^ was utilized to correlate with the amount of wax remnant adhering to tissue section samples. The pixel of WPK-FTIR images presented the distribution of paraplast wax and beeswax remnant, and the data normalized by the number of carbon atoms on human rectal NENs as shown in Fig. 3A and B.

The results of WPK-FTIR imaging of NEN in rectal tissue sections exhibited that more beeswax remnant was observed on the surface of the benign and malignant areas of both NET G1 and NEC G3 than paraplast. More beeswax remnant was observed in the malignant area compared with the benign one as shown in Fig. 3A and B. The areas of malignant tissue section in NET G1 and NEC G3 showed that more beeswax remnant was observed than paraplast wax remnant. Therefore, we proposed that the locations in malignant mucosa had a higher affinity with beeswax than paraplast wax. The result indicated low levels of terminal sialylation in the benign area of NENs^12^. Interestingly, there was a significant difference in the IR absorbance ratio of beeswax-remnant to paraplast -remnant between malignant and adjacent benign areas in NET G1 (p = 0.047). A higher ratio of the beeswax-to-paraplast remnant was noted in the malignant area than that benign mucosa in NET G1; besides, NEN tissue sections at NET G1 showed a relatively higher ratio of beeswax-to-paraplast remnant adhering to tissue sections than that of NEC G3 (p = 0.195), which suggested more terminal sialylation in NET G1. The quantitative results of average adsorption levels of wax in three representative fields are shown in Fig. 3C.

### Neuroendocrine tumor in colonic tissues

Tissue section areas from the benign tissue adjacent to the layer of normal epithelium and malignant lesions were examined from one colonic NET G1 and one colonic NEC G3 tissue samples Fig. 2B and E.

**Figure 4:**
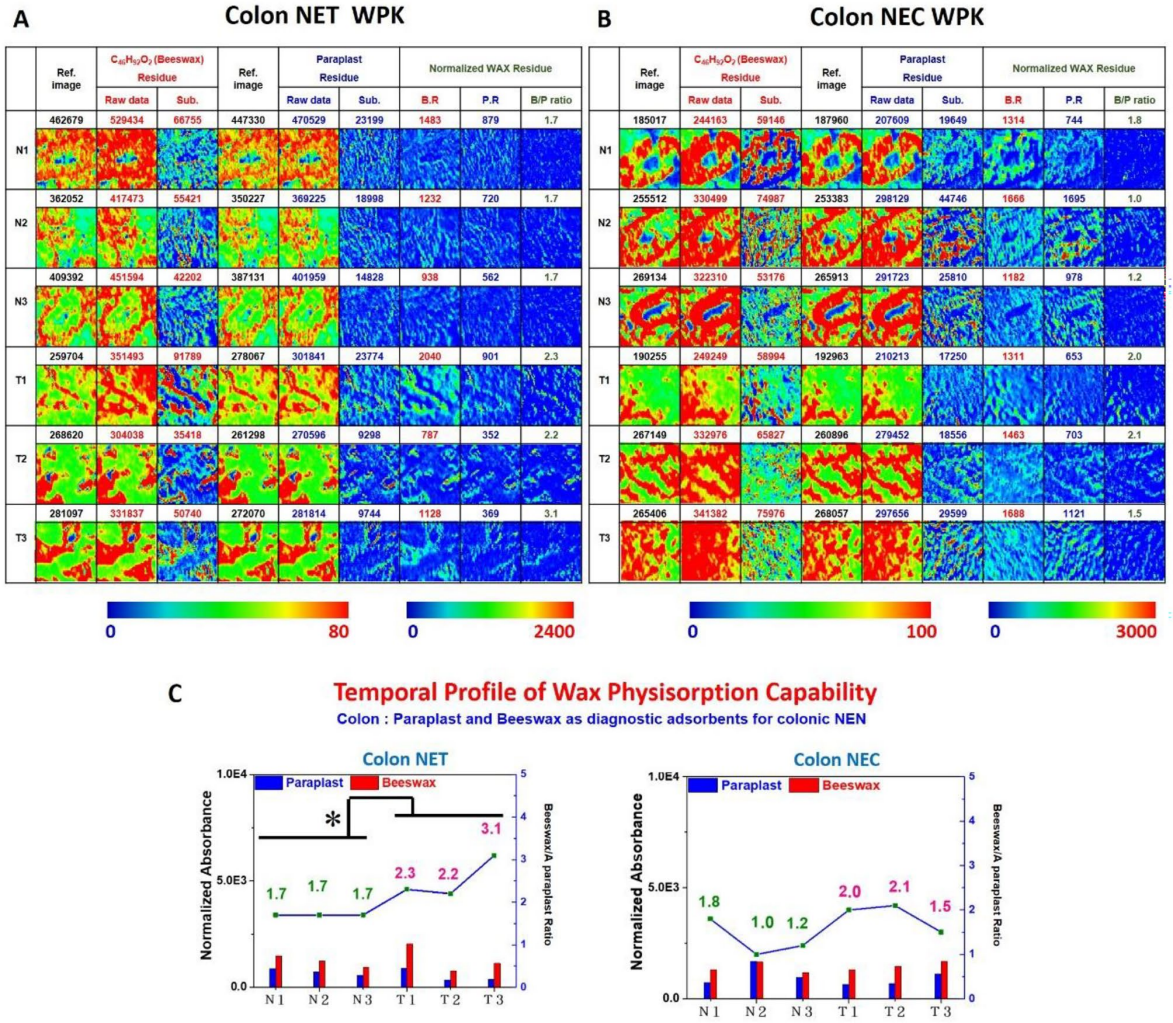
(**A**, **B**) The WPK-FTIR images of the remnant of beeswax and paraplast wax adhering to the surface and (**C**) the quantitative results in colonic NENs. A higher beeswax-remnant to paraplast- remnant attached to the surface of the malignant areas compared to benign areas was found in colonic NET G1 (*p = 0.043).

The lateral-resolved chemical images of wax remnant presented the distribution of paraplast wax and beeswax remnant adhering to human colonic NENs. The results of WPK-FTIR imaging of NEN in colon tissue sections also showed that more beeswax remnant than that of paraplast was observed on the surface of the benign and malignant areas of both NET G1 and NEC G3. The malignant areas exhibited a higher ratio of beeswax-to-paraplast remnant than that of the benign areas shown in Fig. 4A and B, which revealed a higher population of the elongated glycan of glycome including glycoprotein, glycolipid, and proteoglycan attached to the surface of the malignant areas compared to benign areas, especially in colonic NET G1 (p = 0.043). The quantitative results of the averaged wax remnant for three representative areas are presented in Fig. 4C. Moreover, the beeswax-to-paraplast remnant ratio of NEN colonic tissue sections in NET G1 and NEC G3 indicated a relatively higher population of elongated glycan caused by aberrant glycosylation for NET G1 (p = 0.104), which was consistent with an increase in terminal sialylation.

### Neuroendocrine tumor in gastric tissues

**Figure 5:**
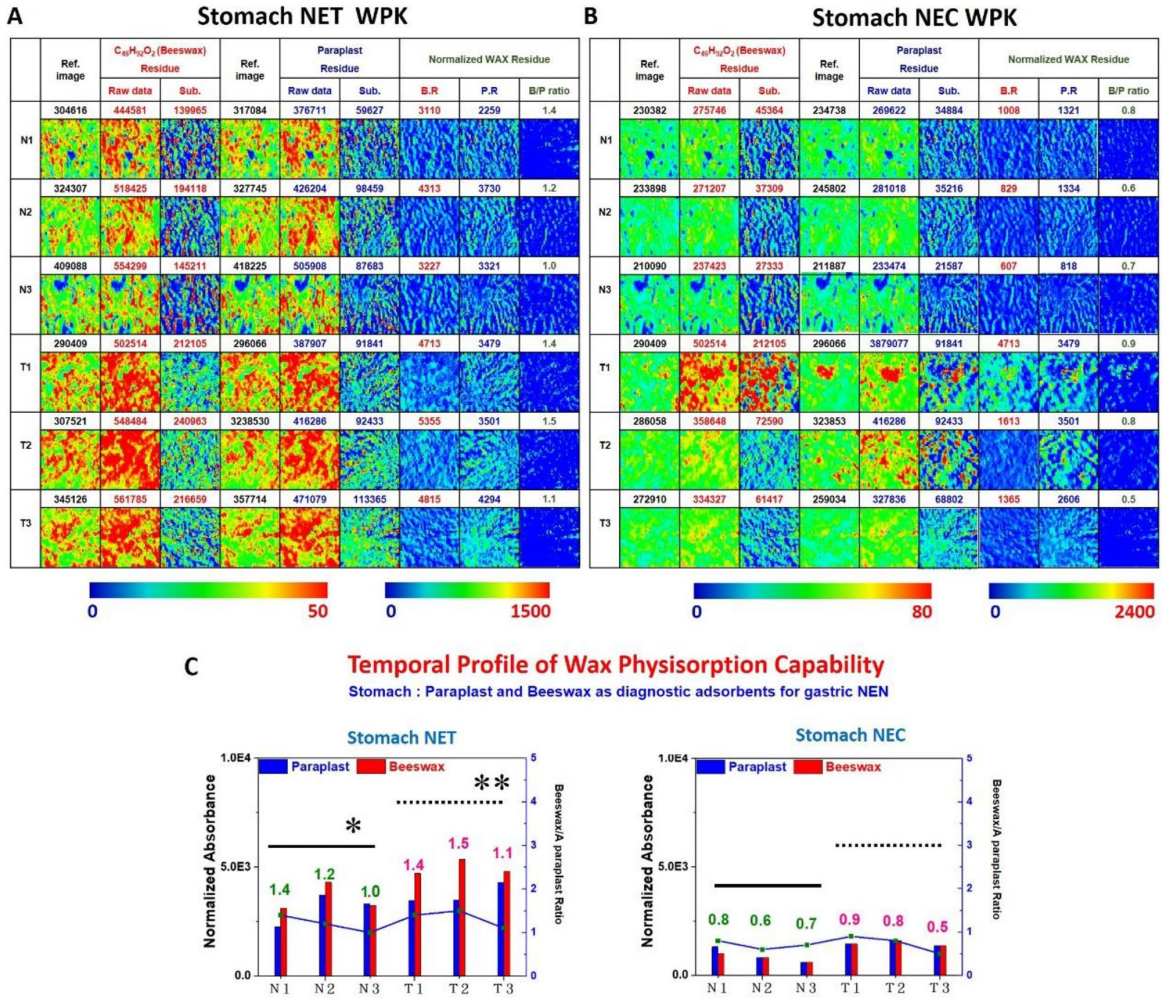
(**A**, **B**) The WPK-FTIR images of the wax remnant and (**C**) the quantitative results in gastric NET and NEC. A significantly higher ratio of the beeswax-to-paraplast remnant of gastric tissue was observed in the benign area of NET G1 compared with NEC G3 (*p = 0.018, solid line). A higher ratio of the beeswax-to-paraplast remnant of tumor cells in NET G1 than that in NEC G3 was also found (**p = 0.024, dotted line).

Human gastric NEN, which were tissue sections of NET G1 and NEC G3 shown in Fig. 2C and F. The spatially-resolved chemical images of gastric NETs tissue sections using WPK-FTIR imaging demonstrated the distribution of paraplast wax remnant and beeswax adhering to the surface of tissue sections illuminated in Fig. 5A and B. More beeswax remnant than paraplast was observed on the surface of benign and malignant areas of NET G1. A significantly higher ratio of the beeswax-to-paraplast remnant of gastric tissue was observed in the benign area of NET G1 compared with NEC G3 (p = 0.018). We also found a higher ratio of the beeswax-to-paraplast remnant of tumor cells in NET G1 than that in NEC G3 (p = 0.024). The quantitative data are shown in Fig. 5C.

## Discussion

Neuroendocrine neoplasm is a well-known hormone-producing neoplasm. The annual incidence in the United States is 3.65 cases per 100,000 population but significant increases in recent years have occurred^13^. They most commonly occur in the GI tract and are also found in the pancreas, lungs, and throughout the body. In multivariate analysis of NEN patients, disease stage, primary tumor site, histologic grade, sex, race, age, and year of diagnosis were independent prognostic markers^14^. Resection is recommended for primary treatment for most NENs. Surgery and local therapies have limited roles in NEC, so radiotherapy combined with chemotherapy will be considered^15^. Usually, patients with grade 3 NECs tended to have more local invasion, multiple distant metastases, and drug resistance to chemotherapy and targeted treatment than grade 1 NETs. To differentiate NEC from NET is crucial for patients’ prognosis, so an alternative diagnostic tool is needed.

As we know, membrane proteins play an important role in signal transduction in carcinogenesis. Currently, therapeutics on cancer membrane-associated proteins has been applied, such as Cetuximab (anti-epidermal growth factor) for colorectal cancer^16^ and Herceptin (anti-Her2neu) for breast cancer^17^. Therefore, drugs targeting cell surface proteins can be used as the main methodology for diagnosis and treatment. FTIR spectroscopy is a state-of-the-art technique, which can be utilized for evaluating molecular biochemical differences between cells and tissues based on the characteristic absorption of functional groups in molecules. Previously, FTIR microspectroscopy was successfully applied to differentiate cancers with respect to disease progression and malignant development^18–20^. Generally, characteristic absorptions of lipids were observed in the spectral range of 3000–2800 cm^−1^. The spectral range of 1630–1690 cm^−1^, and 1520–1560 cm^−1^, were assigned to the amide I band (C=O stretching vibration motion), amide II band (the coupling of C–N stretching vibration and C–N–H bending vibration motion)) of protein respectively^4^. The spectral range of nucleic acids is typically considered between 1800 and 700 cm^−1^^21^, and the wave number between 950 and 1200 is regarded as the fingerprint region for carbohydrates^22^. The FTIR spectrum of wax adsorption showed a similar characteristic in the spectral range of 3000–2800 cm^−1^ to that of the tissue section sample; consequently, the infrared absorption of wax remnant adhering to the surface of the tissue sample section can be estimated by subtracting the lipid absorption before waxing sample. According to molecular structure, paraplast (C.N. 24-27) and beeswax (C_30_H_61_CO_2_C_15_H_31_) are regarded as glycan adsorbents for physical binding regular (short-chain) and elongated (long-chain) glycans of glycoconjugates. Accordingly, WPK-FTIR imaging can be utilized for differentiating normal cells from precursor cancerous change and malignant cells by profiling the chain length of oligosaccharide remnant (glycan) of glycoconjugates on the sample surface.

Based on our findings, a higher ratio of the beeswax-to-paraplast remnant was observed in NET and NEC tumor cells, which indicated a greater affinity with beeswax than paraplast wax. Accordingly, stronger physical adsorption was suggested as occurring on the surface between tissue section of NETs and NECs tumors and wax adsorption caused by length-similarity or one-dimensional geometric length-similarity, resulting in further populating of the elongated glycan of glycoconjugates of protein onto the tissue section surface due to terminal sialylation^12,23^. Furthermore, adjacent benign areas showed less affinity for beeswax with an estimated 46 hydrocarbons chain, indicating lower populating of the elongated glycans glycoconjugates anchored on the surface of the tissue section. NENs and adjacent benign mucosal areas exhibited high selectivity of beeswax and paraplast, respectively. WPK-FTIR imaging seems to be capable of differentiating malignant from adjacent benign lesions at different locations in the GI tract by introducing beeswax-to-paraplast remnant ratio accounting for the populations of long-chain and short-chain glycan on the surface of the tumor tissue section.

Furthermore, a terminal sialylation of glycoconjugates has been demonstrated in the development of advanced cancer^12,23^; consequently, a higher beeswax-to-paraplast remnant ratio in NETs compared with NECs is likely related to aberrant sialylation of glycoconjugates. Accordingly, the more length similarity expected between beeswax and glycan of glycoconjugates of NENs tumors, the greater the physical physisorption expected between wax adsorbent and glycoconjugates on the sample surface due to van der Waals interaction. Chiu et al., reported that the relatively stronger capability of physisorption between the cell surface and beeswax was established by the structure and polarity of glycoconjugates on the cell surface in malignant samples rather than that of normal samples. In contrast, the capability of physisorption between the cell surface and nonpolar paraplast wax of normal tissues appears relatively stronger than that of malignant ones^3^. Certain specific cell surface proteoglycans were also investigated to be associated with NET differentiation^24^. The structural differences of disulfide bones and glycosaminoglycan chains between these proteoglycans could result in variable physisorption for beeswax and paraplast wax^25^.

In the present study, rectal and colonic NEN tissue sections of NET G1 were identified as having a relatively higher beeswax-to-paraplast remnant ratio than that of NEC G3. The Significantly higher ratio of the beeswax-to-paraplast remnant was also observed in selected areas of adjacent benign tissue and tumor cells in gastric NET G1 compared with that in gastric NEC G3. Anatomically, the rectum and colon are both parts of the large intestine, so they share similar physiological phenomenon and WPK-FTIR results undoubtedly. The data of WPK-FTIR in the stomach was not completely consistent with the large intestine which may be influenced by PH level and cell origins. However, the rectum is the most common primary site of GI NENs, followed by the stomach and colon in Taiwan^2^. The data from these three different locations could support that the WPK-FTIR method provides us with a more quantitative alternative for making a diagnosis between NET G1 and NEC G3 beyond being based on morphology. It could improve the differential diagnosis upon specimen limitation or intra-observer variability. Cell membrane glycoconjugates play crucial roles in carcinogenesis, which is consistent with our aim to establish the method of WPK-FTIR imaging to distinguish the difference between low and high-grade NEN tumors by beeswax-to-paraplast remnant ratio.

From a translational medicine perspective, previous literature has demonstrated the different genomic alterations in NETs and NECs. Common mutations in *TP53*, *KRAS,* and *RB* were noted in NECs, and DAXX/ATRX and mTOR mutations were identified in NETs. Recent evidence has indicated that disrupting ceramide glycosylation of the posttranscriptional process can be affected by the p53 gene^26^. In bladder cancer, abnormal protein glycosylation was also investigated as associated with PI3K/Akt/mTOR pathways^27^. The different pathogenesis between NETs and NECs may also cause variable glycosylation and different clinical prognoses in patients with the results suggesting that proteoglycan might be involved in cancer development in a complex manner. Due to tumor-related glycosylation alterations used as the targets for tumor diagnosis and treatment nowadays^28^, we believe that this WPK-FTIR method could provide another novel tool for further diagnosis and therapeutic guidance.

Nonetheless, this study had some limitations. Firstly, it is a retrospective design lacking data regarding tumor size and clinical information. However, all specimens were collected at a single academic medical center, and the reliability of tissue fixation, processing, and diagnostic criteria strengthens our results. The status of tumor metastasis and progression should be further evaluated to predict the clinical behavior of these diseases. Secondly, there were two samples for each location investigated. The two ends of NENs were chosen to explore the differences by using the WPK-FTIR method. The intermediate NET G2 and NET G3 should be considered to enroll and expand the sample size. A further prospective study with available clinical data and more experiment numbers may be able to prove the value of the WPK-FTIR method as an alternative tool in clinical routine.

## Conclusions

In conclusion, WPK-FTIR imaging has been demonstrated as a potential tool in differential diagnosis of grading of NENs according to the physisorption capability between diagnostic wax adsorbents and surface molecules of the tissue section. Similar presentations of WPK-FTIR imaging in variable locations of NENs suggest similar pathogenesis of these tumors. This is the first study to identify the diagnostic role of surface protein-linked glycans residues using WPK-FTIR imaging in NENs. This could be applied as a subjective diagnostic biomarker for clinical therapeutic decision-making.

## Data Availability

The datasets used and/or analyzed during the current study are available from the corresponding author upon reasonable request.
